# N-acetylcysteine amide decreases oxidative stress but not cell death induced by doxorubicin in H9c2 cardiomyocytes

**DOI:** 10.1186/1471-2210-9-7

**Published:** 2009-04-15

**Authors:** Rong Shi, Chuan-Chin Huang, Robert S Aronstam, Nuran Ercal, Adam Martin, Yue-Wern Huang

**Affiliations:** 1 Department of Chemistry, Missouri University of Science and Technology, 400 W. 11^st ^Street, 142 Schrenk Hall, Rolla, MO 65409, USA; 2 Department of Biological Sciences and M S&T cDNA Resource Center, Missouri University of Science and Technology, 400 W. 11^st ^Street, 105 Schrenk Hall, Rolla, MO 65409, USA

## Abstract

**Background:**

While doxorubicin (DOX) is widely used in cancer chemotherapy, long-term severe cardiotoxicity limits its use. This is the first report of the chemoprotective efficacy of a relatively new thiol antioxidant, N-acetylcysteine amide (NACA), on DOX-induced cell death in cardiomyocytes. We hypothesized that NACA would protect H9c2 cardiomyocytes from DOX-induced toxicity by reducing oxidative stress. Accordingly, we determined the ability of NACA to mitigate the cytotoxicity of DOX in H9c2 cells and correlated these effects with the production of indicators of oxidative stress.

**Results:**

DOX at 5 μM induced cardiotoxicity while 1) increasing the generation of reactive oxygen species (ROS), 2) decreasing levels and activities of antioxidants and antioxidant enzymes (catalase, glutathione peroxidase, glutathione reductase) and 3) increasing lipid peroxidation. NACA at 750 μM substantially reduced the levels of ROS and lipid peroxidation, as well as increased both GSH level and GSH/GSSG ratio. However, treating H9c2 cells with NACA did little to protect H9c2 cells from DOX-induced cell death.

**Conclusion:**

Although NACA effectively reduced oxidative stress in DOX-treated H9c2 cells, it had minimal effects on DOX-induced cell death. NACA prevented oxidative stress by elevation of GSH and CYS, reduction of ROS and lipid peroxidation, and restoration of antioxidant enzyme activities. Further studies to identify oxidative stress-independent pathways that lead to DOX-induced cell death in H9c2 are warranted.

## Background

Doxorubicin (Adriamycin^®^) is a potent and broad-spectrum antineoplastic agent used in the treatment of a variety of cancers, including leukemias, lymphomas, and breast, lung, and ovarian cancers [1]. The antitumor mechanism of DOX involves inhibition of both topoisomerase II and DNA synthesis [2]. Unfortunately, long-term treatment with DOX is limited by irreversible cardiomyopathic changes and consequent congestive heart failure. The cardiotoxicity is believed to be caused by the generation of free radicals leading to dysfunction of mitochondria in cardiac cells, interference with cell calcium regulation, and bioenergetic failure [3-5].

The *in vitro *metabolism of DOX by cardiac and liver microsomal membranes includes enzymes such as cytosolic xanthine oxidase, microsomal nicotinamide adenine dinucleotide phosphate (NADPH)-cytochrome P450 reductase, which is present in all tissues (e.g., heart, liver), and mitochondrial cytosolic NADPH dehydrogenase, which is uniquely present in cardiac cells [6,7]. DOX is bio-reduced to a semiquinone free radical that rapidly undergoes 1) further reduction to a hydroquinone, 2) formation of covalent adducts with DNA or proteins, or 3) transfer of the unpaired electron to an electron acceptor [8]. In the presence of oxygen, the semiquinone radical produces O_2_^.-^, which can be converted by superoxide dismutase to hydrogen peroxide (H_2_O_2_). In the presence of reduced iron, H_2_O_2 _is decomposed to the highly toxic hydroxyl radical (HO^.^). Superoxide, H_2_O_2_, and HO^. ^cause peroxidation of unsaturated membrane lipids and induce irreversible tissue damage by inactivating key proteins and enzymes present in the cardiac sarcoplasmic reticulum and in the mitochondrial respiratory chain [9-11].

Considerable attention has been paid to alleviate DOX-induced oxidative stress with DOX by compounds that 1) function as antioxidants or 2) regulate the expression of endogenous antioxidants. For example, carvedilol [12], melatonin and its synthetic derivatives [4,13], metallothionein [14], iron and iron chelators [15] have been tested. To date, dexrazoxane is the only iron chelator that has been approved for reducing DOX-induced cardiotoxicity. However, this chelator also reduces DOX's antineoplastic activity, causes myelosuppression, and may increase the risk of developing secondary malignancies [16,17].

The thiol group plays an important role in biological system. Thiol oxidation can result in protein structure alteration leading to compromise of protein function. The thiol group appearing in a variety of proteins or nonproteins, e.g. glutathione (GSH), undergoes reversible thiol-disulfide interactions to mediate the oxidant-induced stress [18]. The use of biothiols, such as GSH, N-acetylcysteine (NAC), homocysteine, cysteine (CYS), and γ-glutamyl cysteine, to mitigate acute oxidative stress induced by anticancer drugs has long been proposed, though their efficacies have not been fully evaluated. NAC did not provide significant antioxidant effects, presumably due to its low lipid solubility that limits its bioavailability [19]. For instance, NAC at 140 mg/kg body weight failed to prevent acute DOX-induced cardiotoxicity [20]. The carboxyl group in NAC is negatively charged at physiological pH, limiting its ability to cross cell membranes. Recently, N-acetylcysteine amide (NACA), a structural analogue of NAC, was synthesized and evaluated in certain *in vivo *and *in vitro *models. Replacing the carboxyl group with an amide increases lipophilicity, allowing it to cross cell membranes. Two studies have shown that NACA could cross the blood-brain barrier, chelate Cu^2+ ^(which catalyzes free radical formation), scavenge free-radicals, protect red blood cells from oxidative stress, and prevent ROS-induced activation of c-Jun N-terminal protein kinase (JNK), mitogen-activated protein kinase MAPK (p38), and matrix metalloproteinases [21,22].

The ability of NACA to protect cardiomyocytes from DOX-induced toxicity has not been investigated. We hypothesized that NACA would protect H9c2 cardiomyocytes by reducing oxidative stress. Accordingly, we determined the ability of NACA to mitigate the cytotoxicity of DOX in H9c2 cells and correlated these effects with the attenuation of oxidative stress.

## Results

### Cytotoxicity of doxorubicin (DOX) and N-acetylcysteine amide (NACA) on H9c2 cells

**Figure 1:**
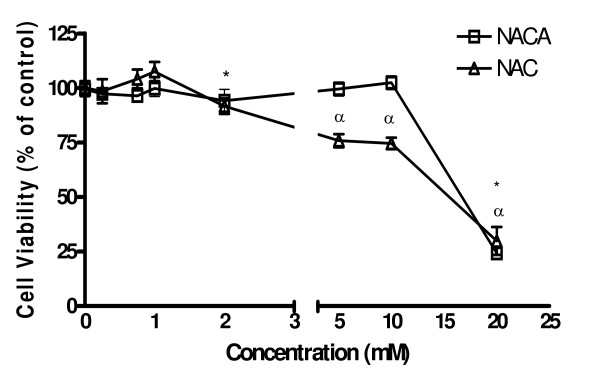
**H9c2 cell viability after 24 h exposure to NACA and NAC at 250 μM, 500 μM, 750 μM, 1 mM, 2 mM, 5 mM, 10 mM, and 20 mM**. Values are mean ± SD from three independent experiments. Significance indicated by: *p < 0.05 NACA vs. control group; ^*α*^*p *< 0.05 NAC vs. control group.

To determine a sublethal concentration of NACA for the study on its ability to protect H9c2 cells from DOX-induced toxicity, we first exposed cells with NACA at 0.25 mM, 0.50 mM, 0.75 mM, 1 mM, 2 mM, 5 mM, 10 mM, and 20 mM for 24 h. A comparative study was conducted on NAC. Both NACA and NAC induced significant cytotoxicity at concentrations ≥ 10 mM and 2.0 mM, respectively. Cell viability was further reduced by 80% at 20 mM of NACA or NAC (*p *< 0.01, n = 5) (Fig. 1). At 1.0 mM of NACA or NAC, the cell viability is comparable to the control group. Thus, we conservatively selected both antioxidants at 0.75 mM in our subsequent studies for comparison purpose.

### Effect of NACA on DOX-induced cytotoxicity

**Figure 2:**
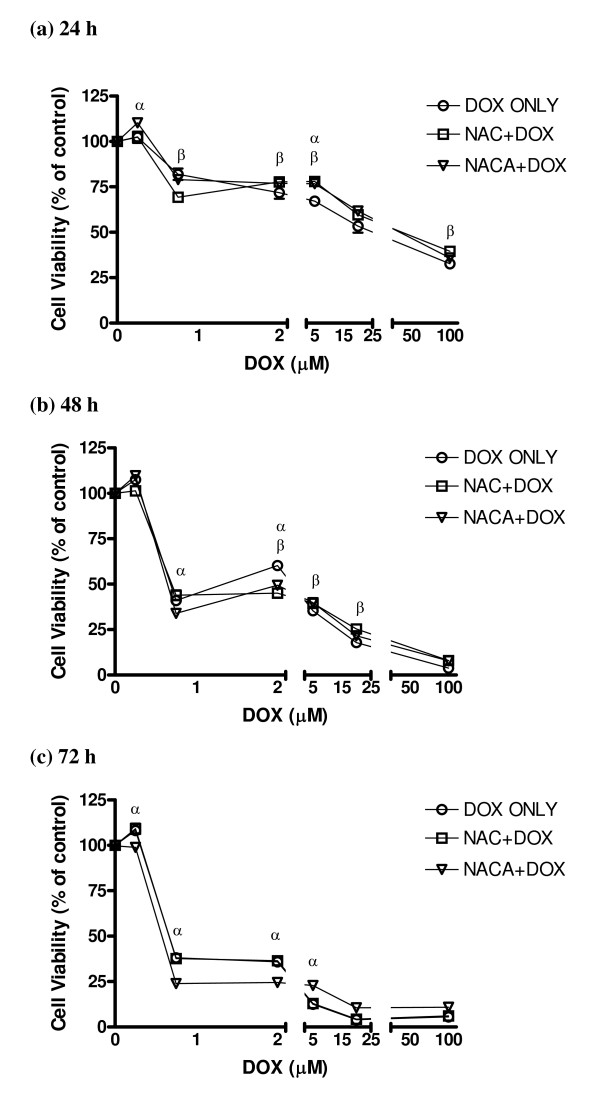
**Effect of NACA and NAC on DOX-induced toxicity in H9c2 cells**. Cells were treated with NACA or NACA at 750 μM for 2 h followed by exposure to freshly prepared cell culture medium with both DOX and NACA or NAC at designated concentrations for 24, 48, or 72 h. The concentrations of DOX were 0.25 μM, 0.75 μM, 2 μM, 5 μM, 20 μM, and 100 μM. Values are mean ± SD from three independent experiments performed in quadruplicate. Significance indicated by: ^*α*^*p *< 0.05 compared between DOX only and NACA + DOX; ^*β*^*p *< 0.05 compared between DOX only and NAC + DOX.

The DOX-induced toxicity was both concentration- and time-dependent in the range of 0.25 – 100 μM. Cell viability at 0.75 μM was reduced by ca. 25%, 60%, and 70% at 24 h, 48 h, and 72 h, respectively (Fig. 2). To study the protective effect of NACA and NAC on DOX-induced toxicity, cells were treated with 750 μM NACA or NAC for 2 h prior to incubation with freshly prepared cell medium containing both DOX and NACA or NAC at designated final concentrations. Both NACA and NAC had minimal protective effects on cytotoxicity at all concentrations of DOX and over a period of 72 h exposure. NACA was most effective at 5 μM of DOX. Accordingly, 5 μM DOX was used in the subsequent studies on oxidative stress.

### DOX-induced oxidative stress in the presence or absence of NACA

**Figure 3:**
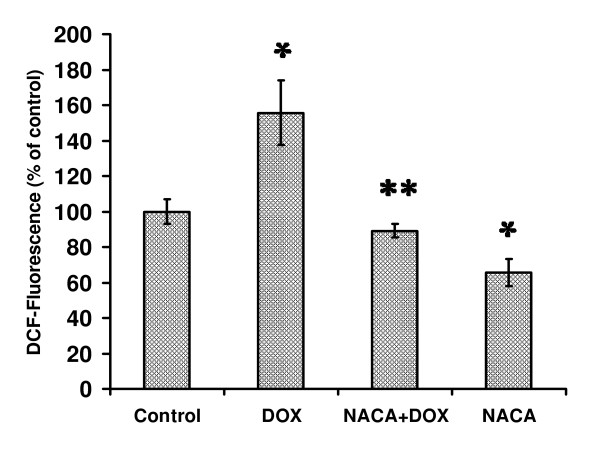
**DCF-fluorescence intensity in H9c2 cells after treatment with DOX in presence or absence of NACA**. Cells were seeded in 24-well plates at a density of 1 × 10^5^cell/well. Cells were treated with or without NACA for 2 h. Then the cell medium was discarded and freshly prepared cell medium containing 5 μM DOX in presence or absence of 750 μM NACA was used to treat the cells for a period of 24 h. The results are presented as percent of the DCF-fluorescence observed in the control group. Values are mean ± SD from three independent experiments performed in quadruplicate. Significance indicated by: *p < 0.05 compared to control group; ** *p *< 0.05 compared to the DOX-only group.

**Figure 4:**
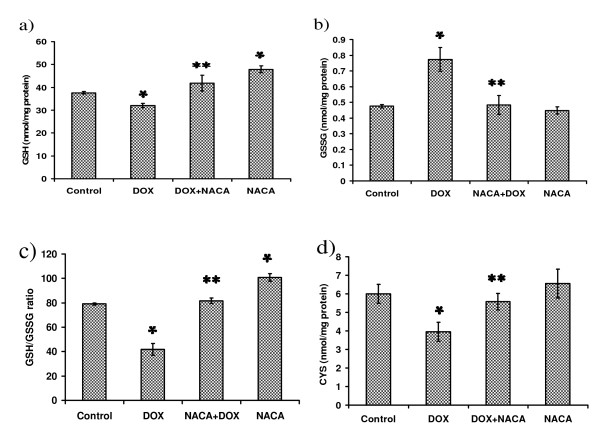
**GSH, CYS, GSSG levels, and GSH/GSSG ratio in H9c2 cells after treatment with DOX in presence or absence of NACA**. H9c2 cells were plated in 75 cm^2 ^tissue culture flasks at a density of 4 × 10^4 ^cells/cm^2^. Cells were treated with or without NACA for 2 h. Then the cell medium was discarded and freshly prepared cell medium containing 5 μM DOX in presence or absence of 750 μM NACA was used to treat the cells for a period of 24 h. Values are mean ± SD from three independent experiments performed in quadruplicate. Significance indicated by: *p < 0.05 compared to control group; ** *p *< 0.05 compared to the DOX-only group.

Treatment with 5 μM DOX increased ROS by 56% compared to the control group (*p *< 0.05, n = 3). The increase was reduced to the control group level in the presence of 750 μM NACA (*p *> 0.05, n = 4) (Fig. 3). The endogenous levels of GSH and CYS were significantly reduced after a 24 h exposure to DOX (*p *< 0.05, n = 4; Fig. 4). Treatment with NACA + DOX resulted in GSH and CYS levels that were higher than those observed in the DOX-only group (*p's *< 0.05, n = 4). Interestingly, in comparison with the control group, the GSH level in the NACA-only group was significantly increased (*p *< 0.05, n = 4), while the CYS levels in the NACA-only group were similar to the control (*p *> 0.4, n = 4).

### NACA restored GSH/GSSG ratio

A change in cellular GSH content is usually accompanied by a concomitant change in the GSSG (the oxidized form of GSH) levels. The GSSG content in the DOX-only group was increased by 61% compared to the control group. NACA reduced the increased GSSG content to the control group level (*p *> 0.05, n = 4; Fig. 4). The GSH/GSSG ratio reflects accumulation of GSSG, thus is a more reliable indicator of cellular redox status. While the ratio was decreased by 47% upon administration of DOX, NACA treatment restored it substantially (*p *< 0.01, n = 4) (Fig. 4). Interestingly, the GSH/GSSG ratio in the NACA-only group was higher than in the control group, suggesting that NACA provides cells with additional cysteine for GSH synthesis (*p *< 0.05, n = 4).

### NACA reduced lipid peroxidation induced by DOX

DOX at 5 μM elevated lipid peroxidation as indicated by the increased detection of cellular TBA-MDA complex. NACA significantly reduced lipid peroxidation compared to the DOX-only group (*p *< 0.05, n = 4) (Table 1). Lipid peroxidation in the NACA-only group did not differ from the control group (p > 0.05, n = 4).

**Table 1:** Lipid peroxidation after DOX and NACA treatment in H9c2 cells.

Group	TBA-MDA (nmol/100 mg protein)
Control	0.92 ± 0.02
DOX (5 μM)	1.55 ± 0.17 *
NACA (750 μM)	0.87 ± 0.04
DOX (5 μM)+ NACA (750 μM)	0.89 ± 0.02**

Significance indicated by: *p < 0.05 compared to control cells; ** *p *< 0.05 compared to DOX-only group.

### NACA restored activities of antioxidant enzymes: catalase (CAT), gluthathione peroxidase (GPx), gluthathione reductase (GR)

CAT activity was 61% lower in the DOX-only group than in the control group (*p *< 0.05, n = 4), while NACA treatment eliminated this reduction (*p *< 0.05, n = 4) (Table 2). DOX reduced GPx activity by 50% compared to the control (*p *< 0.05, n = 4). NACA was capable of fully mitigating the reduction (*p *< 0.05, n = 4) (Table 2). GR activity was 84% lower in DOX-only cells compared to the control group (*p *< 0.01, n = 4), and NACA significantly restored the reduction (*p *< 0.05, n = 4) (Table 2). Activity of CAT, GPx, and GR did not differ between the control group and NACA-only group.

**Table 2:** Antioxidant enzyme activities.

Group	Catalase (mU/mg protein)	GPx (ΔA/min/mg protein)	GR (mU/mg protein)
Control	10.94 ± 2.13	24.77 ± 3.91	5.13 ± 0.09
DOX (5 μM)	4.30 ± 0.45*	12.50 ± 1.74*	0.83 ± 0.04*
NACA (750 μM)	12.85 ± 0.25	21.44 ± 2.39	4.58 ± 0.60
DOX (5 μM)+ NACA (750 μM)	13.13 ± 2.17**	19.31 ± 3.07**	4.22 ± 0.53**

Significance indicated by: *p < 0.05 compared to control cells; **p < 0.05 compared to DOX-only group.

## Discussion

In the present study, DOX significantly reduced cell viability in a concentration- and time-dependent fashion. Though NACA reduced oxidative stress, it had only minimal protective effects on DOX-induced cytotoxicity. Thus, it appears that DOX-induced cell death may have involved redox shift-independent mechanism(s). Previous studies have shown that DOX toxicity can be mediated by the redox-shift dependent pathway as well as by topoisomerase II activation; the latter leads to DNA cleavage, caspase-3 activation and eventually apoptosis [23,24]. The precise contributions of ROS-dependent pathways and the topoisomerase- pathway to DOX-induced cell death remain to be determined.

NACA was capable of restoring GSH, CYS, and GSH/GSSG ratio that were reduced by DOX. NACA supplementation reduced oxidative stress by at least two means. First, NACA may supply cysteine required for GSH synthesis. Second, NACA may convert GSSG to GSH by a non-enzymatic thiol-disulfide exchange. This argument is supported by the increased GSH level observed in the NACA-only group. Our finding on the increased GSH levels in the NACA-only group is in agreement with Offen et al. [22]. Offen *et al*. further suggested that NACA was also able to reduce Cu^2+^-mediated free radical formation, through an undetermined chemical mechanism [22].

NACA prevented the DOX-induced decrease of GR activity. A similar phenomenon of GR activity protection by antioxidants was observed in a study on cadmium-induced oxidative toxicity [25]. The mechanism of restoration remains unclear, though one suggestion has been made. GR-mediated reduction of GSSG to GSH is NADPH-dependent. Regeneration of NADPH from NADP^+ ^requires glucose-6-phosphate dehydrogenase (G6PD). However, G6PD activity may also be reduced by oxidative stress; thus, GR activity might be limited by the G6PD-dependent supply of NADPH. It is possible that NACA prevented DOX-induced decrease of G6PD activity. In the present study, however, G6PD activity was not measured, and additional studies are required to evaluate this mechanism. It should be noted that although GR plays an important role in regenerating endogenous GSH from GSSG, new studies suggested that alternative mechanisms to reduce GSSG and other disulfides may just be enough for animal's normal viability [26]. In addition to the above pathways to increase cellular GSH content, glutathione S-transferase (GST) may conjugate GSH directly to oxidized derivatives of DOX and thus play a crucial role in attenuating the elevated oxidative stress [27].

Increase or activation of endogenous ROS-scavenging antioxidants or enzymes has been shown to protect cells from oxidative damage. For instance, 3*H*-1,2-dithiole-3-thione, a chemoprotective agent, protects against DOX-mediated injury in cardiac cells by inducing cellular antioxidants and enzymes such as GSH, CAT, GPx, GR, and GST [28]. In the present study, the activities of CAT, GPx, and GR in H9c2 cells were significantly decreased following treatment with DOX, but their levels remained at near control values in the presence of NACA, reflecting the restoration of a healthier cellular redox state.

CAT is particularly important in that its relatively low constitutive level in cardiacmyocytes may predispose cardiac muscle to oxidative stress damage [29]. It has been shown that CAT activity can be significantly reduced by DOX [30,31]. In the present study, NACA was able to prevent the loss of CAT activity caused by subsequent to DOX.

Cardiac muscle is very susceptible to oxidative damage due in part to the rapid inactivation of GPx [29]. Overexpression of GPx in endothelial cells and myocytes significantly decreases DOX-induced NF-kB activation which leads to apoptosis [32]. In the present study, we found that the enzymatic activity of GPx, which utilizes GSH as a substrate to reduce H_2_O_2 _to H_2_O, is decreased by DOX. This fall in GPx activity might be a result of the decrease in GSH, as suggested by others [33]. The increased intracellular GSH content in the NACA group might activate the GSH-dependent GPx, thereby preventing the accumulation of H_2_O_2_. Thus, the ability of NACA to reactivate GPx activity in our study is further evidence of cardioprotection.

## Conclusion

Though NACA was able to provide oxidative relief, it only had minimal protective effect on DOX-induced cytotoxicity. NACA prevented oxidative stress by elevation of GSH and CYS, reduction of ROS and lipid peroxidation, and restoration of antioxidant enzyme activities. Further studies to identify various mechanisms of cytotoxicity and their inter-relationships, if any, which lead to DOX-induced cell death are warranted.

## Methods

### Chemicals and reagents

N-(1-pyrenyl)-maleimide (NPM) was purchased from Aldrich (Milwaukee, WI, USA). N-acetylcysteine amide (NACA) was obtained from David Pharmaceuticals (New York, NY, USA). HPLC grade solvents were purchased from Fisher Scientific (Fair Lawn, NJ, USA). All other chemicals were purchased from Sigma (St. Louis, MO, USA).

### Cell culture and toxicity studies

H9c2 cells, a clonal line of cardiomyocytes derived from embryonic rat heart tissue, were purchased from the American Type Culture Collection (ATCC, Manassas, VA, USA). H9c2 cells exhibit many of the properties of cardiac muscle, including electrophysiological activity, ion channels, and autonomic receptors [34]. This cell line has been previously used in doxorubicin-related cytotoxicity studies [35,36].

Following the protocol provided by ATCC, the cells were cultured in a complete medium consisting of Dulbecco's modified Eagle's medium (DMEM) supplemented with 10% fetal bovine serum (FBS), 4 mM L-glutamine adjusted to contain 1.5 g/L sodium bicarbonate and 4.5 g/L glucose, and 1% (v/v) penicillin and streptomycin. The cultures were maintained at 37° in a 5% CO_2 _humidified atmosphere. Cells were subcultured at a 1:4 ratio every 3 days using 75 cm^2 ^tissue culture flasks.

After termination of experiments, a serine borate buffer (SBB) was used during cell homogenate preparation to prevent potential oxidation of biothiols in GSH, CYS, and GSSG analyses, and of in the lipid peroxidation assay. The SBB buffer contains 100 mM Tris-HCl, 10 mM borate, 5 mM serine, and 1 mM diethylenetriaminepentacetic acid with the final pH adjusted to 7.0 using concentrated NaOH. The cell samples are homogenized in the SBB buffer with a tissue homogenizer (Model 985-370, type 2, Biospec Products, Inc.) on ice for 2 min, with 5 s intervals of homogenization.

### Cytotoxicity of DOX in H9c2 cells

To choose a sublethal concentration of NACA and NAC for the study on their ability to protect cells from DOX-induced toxicity, we first exposed H9c2 cells with NACA or NAC at 0.25 mM, 0.50 mM, 0.75 mM, 1 mM, 2 mM, 5 mM, 10 mM, and 20 mM for 24 h. Untreated cells were used as the control for each experiment. At 1.0 mM of NACA or NAC, the cell viability did not differ from the control group. We conservatively selected a nontoxic concentration, 0.75 mM, for both antioxidants for the subsequent experiments.

To determine effectiveness of NACA and NAC in protection of H9c2 cells from DOX-induced toxicity, cells were treated with NACA or NAC at 0.75 mM for 2 h followed by exposure to freshly prepared cell culture medium with DOX in presence or absence of NACA or NAC at designated concentrations. The concentrations of DOX were 0.25 μM, 0.75 μM, 2 μM, 5 μM, 20 μM, and 100 μM. The exposure durations were 24 h, 48 h, or 48 h. Cells incubated with NACA or NAC alone were used as the control.

### MTS assay

The MTS bioreduction assay (Cell Titer 96^® ^Aqueous One Solution Assay, Promega) is an assay based on the bioreduction of 3-(4,5-dimethyl thiazol-2-yl)-5-(3-carboxymethoxy phenyl)-2-(4-sulfophenyl)-2H-tetrazolium (MTS) by viable cells to a colored formazan product that is soluble in culture media [37]. Absorbance at 490 nm is proportional to the number of living cells in the culture.

### Intracellular ROS measurement

Intracellular ROS generation was measured using a well characterized probe, 2', 7'-dichlorofluorescin diacetate (DCFH-DA) [38]. DCFH-DA is hydrolyzed by esterases to dichlorofluorescin (DCFH), which is trapped within the cell. This nonfluorescent molecule is then oxidized to fluorescent dichlorofluorescin (DCF) by action of cellular oxidants. After exposure to 5 μM DOX, with or without treatment with 750 μM NACA, the cells (10^6^/ml) were incubated in 2 ml of 140 mM NaCl, 5 mM KCl, 1 mM MgCl_2_, 5.6 mM glucose, 1.5 mM CaCl_2_, and 20 mM Hepes-Na, pH 7.4, and allowed to take up 5 μM DCFH2-DA at 37°C for 20 min in an atmosphere of 95% air and 5% CO_2_. After loading samples on 96 well plates, DCF fluorescence was measured at 485 nm excitation and 520 nm emission.

### GSH and CYS measurement

Cellular levels of GSH and cysteine were determined by HPLC [39]. Cells were seeded at a density of 4 × 10^4 ^cells/cm^2 ^on 75 cm^2 ^flasks, and the flasks were incubated for 24 h with fresh medium containing DOX (5 μM), or DOX (5 μM) + NACA (750 μM), or NACA (750 μM) at 37°C. Following the incubation period, the cells were removed and homogenized. Twenty μl of diluted cell homogenate were added to 230 μl of serine borate buffer and 750 μl of NPM (1 mM in acetonitrile). NPM reacts with free sulfhydryl groups to form fluorescent derivatives which yield fluorescent adducts that can be detected fluorimetrically (λex = 330 nm, λem = 376 nm). After incubation at room temperature for 5 min, the samples were acidified with 10 μl of 2 N HCl to stop the reaction. The derivatized samples were filtered through a 0.45 μm acrodisc and then injected onto the HPLC column.

### Glutathione disulfide (GSSG) measurement

H9c2 cells were seeded in 75 cm^2 ^tissue culture flasks at a density of 4 × 10^4 ^cells/cm^2^. The cells were treated for 24 h with 5 μM DOX, 750 μM NACA, or 5 μM DOX + 750 μM NACA. Untreated cells served as controls. Sixteen μl of 6.25% 2-vinylpyridine in absolute ethanol were added to 84 μl of cell homogenate. This suspension was incubated at room temperature for 60 minutes to block the thiol group of the GSH already present. NADPH (95 μl of 2 mg/ml) in nanopure water and 5 μl of 2 units/ml glutathione reductase were added to reduce GSSG. An aliquot of 100 μl of the treated samples was diluted with 150 μl H_2_O, and then immediately derivatized with 750 μl of 1.0 mM NPM. Fluorescence was then measured (λex = 330 nm and λem = 376 nm) (Thermo Electron Corp., Austin, TX, USA).

### Lipid peroxidation measurement

Malondialdehyde (MDA) is a thiobarbituric acid reactive substance (TBAR). The extent of cellular lipid peroxidation was determined by measuring concentrations of TBA-MDA complex. Cell homogenate (350 μl), 100 μl of 500 ppm butylated hydroxytoluene, and 550 μl of 10% trichloroacetic acid were combined, and the suspension was boiled for 30 min. An aliquot (500 μl) of the supernatant was removed and 500 μl of thiobarbituric acid added. From this solution, 500 μl was removed and added to 1.0 ml of n-butanol. This mixture was vortexed, and centrifuged for 5 min at 110 × g to facilitate phase separation. Fluorescence was then measured (λex = 515 nm and λem = 550 nm) [40].

### Measurement of antioxidant enzymes

H9c2 cells were plated in 75 cm^2 ^tissue culture flasks at a density of 4 × 10^4 ^cells/cm^2 ^and allowed to attach for 24 h in complete cell culture medium. The cells were then divided into four groups for 24 h treatment, as follows: 1) control, 2) 5 μM DOX, 3) 5 μM DOX + 750 μM NACA, and 4) 750 μM NACA. Assays for antioxidant enzymes were then carried out.

### Catalase (CAT) activity assays

Catalase activity was determined spectrophotometrically as described by Aebi [41]. Cell homogenates were diluted with 50 mM phosphate buffer (pH = 7.0). The exponential decrease of 10 mM hydrogen peroxide was measured at 240 nm in the presence of cell homogenate. Reaction mixtures without cell homogenate were used as tissue blanks.

### Glutathione peroxidase (GPx) activity determination

Glutathione peroxidase activity was determined by the method of Paglia and Valentine [42]. Cell homogenates were diluted with phosphate buffer (pH 7.6). Tert-butyl hydroperoxide was first reduced by glutathione peroxidase and then recycled by glutathione reductase coupled with nicotinamide adenine dinucleotide phosphate (NADPH) oxidation. The rate of decrease in NADPH was monitored spectrophotometrically at 340 nm as a measure of GPx activity.

### Glutathione reductase (GR) activity determination

GR activity was determined using the method of Carlberg and Mannervik [43]. Glutathione reductase is required for the NADPH-dependent conversion of oxidized glutathione (GSSG) to reduced glutathione (GSH). Cell homogenates were diluted with phosphate buffer (pH = 7.5). The exponential decrease of NADPH was measured spectrophotometrically at 340 nm in the presence of cell homogenate. Reaction mixtures in which potassium phosphate buffer was used instead of glutathione disulfide in phosphate buffer were used as blanks.

### Protein determination

Protein levels were determined by the Bradford method with Coomassie blue (Bio-Rad) [44]. Two and a half millimeters of the diluted reagent were added to 0.05 ml of a bovine serum albumin standard solution containing 10 to 100 mg/ml protein. This mixture was incubated at room temperature for 5 to 10 minutes and the optical density was measured at 595 nm.

### Statistical analysis

The data are presented as the mean ± SD. One-way analysis of variance (ANOVA) and Student-Newman-Keuls multiple comparison tests were used to analyze the significance of the differences between treatment groups. Statistical significance is set at 0.05.

## Competing interests

The authors declare that they have no competing interests.

## Authors' contributions

We suggest the following kind of format (please use initials to refer to each author's contribution): RS carried out the measurement of antioxidants and antioxidant enzymes. CH carried out the cell viability curves and statistical analysis. RSA and AM participated in the design of the study. YH and NE conceived of the study, and participated in its design and coordination and helped to draft the manuscript. All authors read and approved the final manuscript.
